# Systemic treatment type is not associated with abnormal post-treatment noninvasive liver stiffness measurement in psoriasis

**DOI:** 10.3389/fimmu.2024.1487959

**Published:** 2024-12-11

**Authors:** Yue Xiao, Jingya Gao, Yiyi Wang, Dan Hao, Wei Yan, Dingke Wen, Siyi Zeng, Shiqi Yang, Yingyu Shi, Wei Li

**Affiliations:** ^1^ Department of Dermatology and Venereology, West China Hospital, Sichuan University, Chengdu, China; ^2^ Department of Neurosurgery, West China Hospital, Sichuan University, Chengdu, China; ^3^ West China School of Medicine, Sichuan University, Chengdu, China; ^4^ Department of Ultrasound, West China Hospital, Sichuan University, Chengdu, China

**Keywords:** BMI, biologics, liver stiffness measurement (LSM), noninvasive assessment, psoriasis, risk factor, sound touch elastography

## Abstract

**Background:**

Psoriasis is commonly associated with metabolic dysfunction-associated steatotic liver disease, raising concerns about the hepatic effects of systemic treatments on psoriasis and its comorbid conditions. This study evaluates liver stiffness measurement (LSM) alterations and identifies predictors of abnormal LSM in psoriatic patients following systemic treatments, including biologics and methotrexate.

**Methods:**

This prospective cohort study is based on the PSOWCH database (Psoriasis Cohort of West China Hospital). We initially included psoriatic patients who had undergone sound touch elastography (STE), then recruited patients who had STE before systemic treatment and reassessed them after at least six months. Three treatment subgroups were formed (interleukin inhibitors, tumor necrosis factor inhibitors, and methotrexate), classifying post-treatment STE outcomes using threshold values of 6.5 kPa and 10.3 kPa.

**Results:**

Among the 52 recruited patients, overall STE values significantly increased during follow-up. Univariate regression analysis showed that age, gender, psoriasis severity, psoriatic arthritis status, and current treatment type were not significantly correlated with abnormal STE outcomes at cutoff values of 6.5 kPa and 10.3 kPa. In the multivariate model, body mass index (BMI) was identified as a risk factor for post-treatment STE ≥ 6.5 kPa (odds ratio [OR], 1.26; 95% CI, 1.04 to 1.60, P=0.031).

**Conclusions:**

This exploratory study reveals that systemic treatment type is not associated with abnormal post-treatment LSM. However, a significant association exists between BMI and abnormal LSM outcomes. These findings highlight the critical importance of BMI management in therapeutic interventions for psoriasis.

## 1 Introduction

Psoriasis is an immune-mediated systemic disorder associated with a range of comorbidities, including metabolic dysfunction-associated steatotic liver disease (MASLD), previously termed nonalcoholic fatty liver disease (NAFLD), which arises in the absence of other identifiable causes such as alcohol consumption, viral infection, or autoimmune conditions (1–5). MASLD is characterized by excessive hepatic fat accumulation resulting from metabolic dysfunction or inflammation (4, 6). This condition could progress to metabolic dysfunction-associated steatohepatitis (MASH), with or without accompanying fibrosis, ultimately leading to liver failure and the potential development of hepatocellular carcinoma (7). Among individuals with MASLD, the severity of liver fibrosis emerges as the most critical risk factor for liver-related morbidity and mortality (8). Epidemiological data indicate that MASLD affects over a quarter of the population in Asian countries, with individuals diagnosed with psoriasis facing an elevated risk for developing fatty liver disease, as well as progressive liver stiffness, compared to the general population (9, 10). This underscores the need for routine monitoring of liver health in patients with psoriasis.

While liver biopsy remains the gold standard for the diagnosis and stratification of liver fibrosis, its clinical application for routine monitoring is constrained by its invasive nature (4, 11, 12). Consequently, noninvasive liver stiffness measurement (LSM) techniques have been increasingly adopted as alternative assessment methods (13). The utilization of LSM in subjects with MASLD has been previously characterized (14, 15). However, research on LSM dynamics, specifically within the context of psoriasis, is notably limited. Regarding the risk of liver stiffness progression in psoriatic patients, concerns have been raised about the hepatotoxicity associated with systemic treatments, in particular, methotrexate (MTX) (9). Furthermore, a significant gap exists in the literature regarding the potential hepatic implications of biologic therapies in this patient population.

Sound touch elastography (STE) is a novel shear wave elastography method that provides real-time imaging of tissue stiffness in the region of interest, potentially allowing for accurate stiffness estimation (16–18). This study aims to utilize STE to investigate longitudinal changes in LSM among psoriasis patients receiving systemic treatments and to identify potential predictors of abnormal LSM outcomes.

## 2 Methods

### 2.1 Data source and participants

This prospective cohort study is based on the PSOWCH database (Psoriasis Cohort of West China Hospital). The establishment of the PSOWCH database occurred in two phases. Initially, we identified psoriatic patients who visited the Department of Dermatology at West China Hospital from January 1, 2010, to December 31, 2020, through a comprehensive review of medical records within the hospital information system. Following this, we commenced prospective enrollment and data collection for patients diagnosed with psoriasis beginning on January 1, 2021. In this study, participants underwent sound touch elastography (STE) reassessment between March 16, 2021, and April 26, 2023. The PSOWCH database received ethical approval from the Biomedical Research Ethics Committee of the West China Hospital, Sichuan University (approval number: 2021-581) and adheres to the ethical standards outlined in the Declaration of Helsinki, as amended in 2013. Informed consents were obtained from all patients enrolled prospectively in the study.

Eligibility inclusion criteria for this study required patients to be over 18 years old without sex limitations, with a diagnosis of plaque psoriasis (ICD-10 code L40.0). Patients were required to have undergone a comprehensive baseline clinical assessment, laboratory testing, and STE examinations. We excluded patients who: (i) had active viral hepatitis infections (hepatitis B or C); (ii) consumed more than 15 grams of alcohol per day on average; (iii) had diagnosed autoimmune liver diseases; (iv) suffered from severe liver or other organ dysfunctions; (v) had hematological or solid tumors; or (vi) were pregnant or lactating.

### 2.2 Follow-up and data collection

From this cohort, we recruited psoriasis patients who completed STE evaluation prior to initiating systemic monotherapy. Following a minimal follow-up period of six months, STE was reexamined. We collected demographic information (age, sex, BMI, etc.), psoriasis-related information (psoriasis duration, the presence of psoriatic arthritis[PsA], prior treatments, and current treatment regimens), alongside laboratory and STE results. We adopted 23 kg/m² and 27.5 kg/m² as BMI cutoffs for overweight and obesity, respectively, based on the WHO’s recommendations for the Chinese population (19). Patients were classified into three treatment subgroups: interleukin (IL) inhibitors, tumor necrosis factor (TNF) inhibitors, and MTX.

### 2.3 STE examination methods

STE was performed using the Resona 7 ultrasound system (Mindray Medical Solutions, Shenzhen, China). Two sonographers, each with over ten years of experience in abdominal ultrasound and eight years in elastography, conducted the examinations. In accordance with the guidelines established by the European Federation of Societies for Ultrasound in Medicine and Biology (EFSUMB), patients were positioned appropriately to facilitate accurate liver stiffness measurements (LSM) on the right liver lobe (20). Liver stiffness via STE was classified into three categories: normal (<6.5kPa), indicative of liver fibrosis (6.5-10.3kPa), and significant liver stiffness suggestive of liver cirrhosis (>10.3kPa). STE’s cutoff values (6.5 and 10.3 kPa) are based on the reference range specified in the examination report.

### 2.4 Statistical analysis

Categorical variables were expressed as counts and percentages, with Fisher’s exact test employed to assess differences across treatment groups. Normally distributed continuous variables were presented as the means and standard deviations (SD) and compared using Student’s t-test. Non-parametric statistics were used for median and interquartile range (IQR) comparisons, applying the Mann-Whitney test. The comparison of STE values between baseline and follow-up was performed using the Wilcoxon signed-rank test due to the non-normal distribution. GraphPad Prism (version 10.11) generated the paired t-test and its accompanying figure.

To identify variables linked to the outcome of abnormal STE results—defined by the threshold values of 6.5 kPa or 10.3 kPa—univariate logistic regression models were initially employed. Considering the study’s exploratory nature and the limited sample size, variables with a *P* value less than 0.1 in the univariate analysis were considered potential factors. Pearson’s correlation coefficient was calculated to assess linear relationships between continuous variables, with collinear variables being excluded from further analysis. Relevant variables were then incorporated stepwise into a multivariate regression model, adhering to an event-per-variable ratio of five. This model was subsequently validated for stability. *P*-values less than 0.05 were deemed statistically significant. All remaining statistical analyses were performed using R software (version 4.3.2).

## 3 Results

### 3.1 Demographic and clinical characteristics

In the PSOWCH, a total of 296 psoriatic patients were screened for STE assessments at West China Hospital, Sichuan University, between January 1st, 2018, and August 1st, 2022. Following the criteria, we excluded fifteen patients with active hepatitis viral B infection. Among these, 52 eligible participants were undergoing systemic monotherapy, which included IL inhibitors (n=27), TNF inhibitors (n=15), and MTX (n=10). There are 21 (40.38%) PsA patients in total, and 7 (25.93%), 10 (66.67%), and 4 (40.00%), respectively, in the three subgroups. All patients were monitored for at least six months before STE reassessment. The demographic and clinical characteristics of the 52 participants are detailed in **Table 1**, revealing baseline differences among the three treatment subgroups in terms of psoriasis duration, proportion of PsA, prior treatment, and other relevant variables.

**Table 1:** Baseline and clinical characteristics of psoriatic patients who examined sound touch elastography.

Variables	IL-group^§^	TNF-group^§^	MTX-group	Total	*P* value
	n=27	n=15	n=10	n=52	
**Age, years, mean (SD)**	41.95 (8.98)	45.30 (12.04)	46.80 (12.88)	43.85 (10.70)	0.39
**Gender (male: female)**	21 (77.78)	12 (80.00)	8 (80.00)	41 (78.85)	0.99
**Height, cm, mean (SD)**	167.74 (7.65)	167.87 (6.33)	164.70 (10.01)	167.20 (7.75)	0.54
**Weight, kg, mean (SD)**	72.37 (10.49)	72.17 (12.26)	68.70 (9.94)	71.61 (10.81)	0.65
**BMI†, kg/m2, mean (SD)**	25.78 (3.85)	25.51 (3.34)	25.29 (2.54)	25.61 (3.44)	0.92
BMI<23	5 (18.52)	4 (26.67)	2 (20.00)	11 (21.15)	0.79
Overweight (23≤BMI<27.5)	16 (59.26)	7 (46.66)	7 (70.00)	30 (57.70)	
General obesity (27.5≤BMI)	6 (22.22)	4 (26.67)	1 (10.00)	11 (21.15)	
Smoking status					
Never smoke, n (%)	18 (66.67)	7 (46.67)	7 (70.00)	32 (61.54)	0.42
Ex-smoker and current smoker, n (%)	9 (33.33)	8 (53.33)	3 (30.00)	20 (38.46)	
Drinking status					
Never drink, n (%)	19 (70.37)	9 (60.00)	7 (70.00)	35 (67.31)	0.72
Drink alcohol, n (%)	8 (29.63)	6 (40.00)	3 (30.00)	17 (32.69)	
Psoriasis status					
Age of onset, years, mean (SD)	28.80 (9.10)	25.90 (8.06)	33.29 (9.57)	28.83 (9.09)	0.14
Duration of psoriasis, years, mean (SD)	13.15 (7.01)	19.40 (9.16)	13.50 (9.08)	15.02 (8.41)	0.054
PASI, median (IQR)	7.20 (7.40)	21.00 (9.16)	11.50 (9.08)	6.75 (6.75)	0.1
BSA, %, median (IQR)	8.00 (13.50)	7.00 (9.00)	4.50 (6.75)	8.00 (10.75)	0.2
PsA, n (%)	7 (25.93)	10 (66.67)	4 (40.00)	21 (40.38)	**0.038**
Prior treatment^‡^					
MTX, n (%)	14 (51.85)	7 (46.67)	10 (100.00)	31 (59.62)	0.011
Acitretin, n (%)	11 (40.74)	6 (40.00)	4 (40.00)	21 (40.38)	0.99
Phototherapy, n (%)	13 (48.15)	9 (60.00)	1 (10.00)	23 (44.23)	**0.033**
Current therapy					
The duration, years, median (IQR)	1.21 (0.72)	1.85 (1.02)	0.82 (0.45)	1.16 (0.98)	**0.0029**
Baseline laboratory examinations					
Alanine aminotransferase, median (IQR)	26 (24)	27 (22.5)	49 (22.25)	29.50 (29.25)	**0.023**
Aspartate aminotransferase, median (IQR)	22 (8.5)	26 (11)	37 (21.25)	25.50 (13.50)	**0.023**
Albumin, mean (SD)	47.19 (2.03)	46.44 (2.26)	45.79 (3.24)	46.70 (2.38)	0.26
Low Density Lipoprotein, mean (SD)	3.12 (0.67)	3.21 (1.02)	2.49 (1.28)	3.08 (0.87)	0.26
High Density Lipoprotein, median (IQR)	1.00 (0.51)	1.23 (0.38)	1.00 (0.28)	1.02 (1.47)	0.64
Total cholesterol, mean (SD)	4.98 (0.81)	5.12 (1.36)	5.10 (1.35)	5.04 (1.05)	0.92
Triglyceride, median (IQR)	1.64 (1.26)	1.48 (1.24)	1.79 (4.87)	1.62 (1.45)	0.60
Uric acid, median (IQR)	403.00 (69.00)	348.00 (100.50)	364.50 (228.25)	385.50 (128.70)	0.39
γ-glutamyl transpeptadase, median (IQR)	24.00 (14.00)	30.00 (60.50)	28.50 (59.50)	26.00 (25.75)	0.35
Glucose, mean (SD)	5.31 (0.67)	5.15 (0.63)	4.94 (0.45)	5.21 (0.63)	0.38
Hemoglobin, median (IQR)	154.0 (14.00)	149.0 (22.00)	150.0 (27.00)	154.0 (26.00)	0.23
Platelets, mean (SD)	208.9 (66.31)	222.2 (60.43)	203.7 (47.09)	211.9 (60.93)	0.73
White blood cells, mean (SD)	7.37 (1.95)	8.23 (1.83)	6.34 (1.58)	7.44 (1.93)	0.062
Baseline STE					
The diagnosis of fatty liver, n (%)	20 (74.07)	11 (73.33)	7 (70.00)	38 (73.08)	0.99
Baseline STE value, kPa, median (IQR)	5.60 (1.00)	5.40 (1.45)	6.15 (0.85)	5.65 (1.2)	0.34
Baseline STE value<6.5 kPa, n (%)	23 (85.19)	12 (80.00)	8 (80.00)	43 (82.69)	0.93
The Baseline STE values≥ 6.5 kPa, n(%)	4 (14.81)	3 (20.00)	2 (20.00)	9 (17.31)	
Re-examine STE					
Re-examine STE value, kPa, median (IQR)	6.8 (2.26)	6.6 (1.71)	7.15 (3.93)	6.80 (2.46)	0.95
Re-examine STE value <6.5 kPa, n (%)	13 (48.15)	7 (46.67)	4 (40.00)	24 (46.15)	0.93
Re-examined STE values ≥ 6.5 kPa, n(%)	14 (51.85)	8 (53.33)	6 (60.00)	28 (53.85)	

Categorical variables expressed as n (%), and continuous variables as mean (standard deviation) or median (iqr). † The BMI classification is according to the suggestions for Chinese population from WHO. ‡The total percentage of previous treatment is over 100% as some of the patients previously has received more than one treatment. §Anti-TNFi included adalimumab (14 patients), Etanercept (1 patient). Anti-interleukin inhibitors included secukinumab (25 patients), ixekizumab (1 patient), guselkumab (1 patient). BMI, body mass index; BSA, body surface area; HBV, hepatitis B virus; HCV, hepatitis C virus; HIV, human immunodeficiency virus; IL, interleukin; MTX, methotrexate; PASI, Psoriasis Area Severity Index; PsA, psoriatic arthritis; STE, sound touch elastography; TNF, tumor necrosis factor.

The bold values are for P values <0.05.

### 3.2 Longitudinal changes in STE values

**Figure 1:**
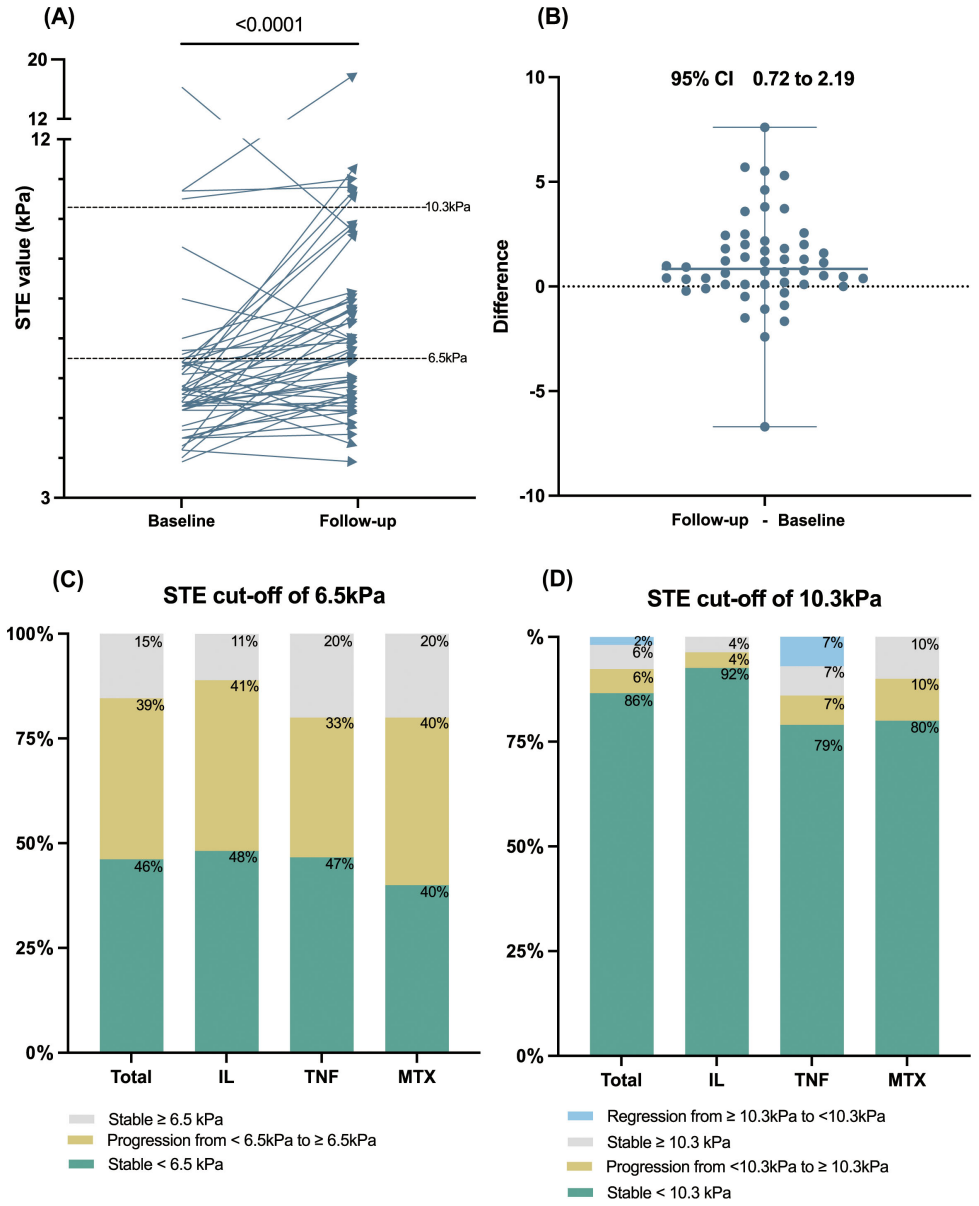
The longitudinal change of STE value among patients with psoriasis during follow-up. **(A)** The STE values significantly elevated after over one year of psoriatic systemic monotherapy (n=52). **(B)** The differences plot of the STE value change. **(C, D)** Percentage of patients with stable, regressed, or progressed STE at a cutoff value of 6.5 kPa or 10.3 kPa, displayed for the total cohort and IL, TNF, and MTX subgroups. STE, sound touch elastography; IL, interleukin; TNF, tumor necrosis factor; MTX, methotrexate.

The primary objective of our study was to evaluate post-treatment alterations in STE values. The baseline STE values for these 52 patients, represented as medians with IQRs, did not significantly differ from the initial cohort of 281 patients [5.65 (1.2) kPa vs. 5.50 (1.6) kPa, *P*=0.47]. Upon reassessment, the post-treatment STE values of the 52 patients demonstrated a statistically significant increase to 6.8 (2.46) kPa after a median follow-up duration of 1.16 (0.98) years (*P*<0.0001). (**Figures 1A, B**) The post-treatment STE values for patients receiving IL inhibitors, TNF inhibitors, and MTX were recorded at 6.8 (2.26) kPa, 6.6 (1.71) kPa, and 7.15 (3.93) kPa, respectively, with no significant discrepancies observed among these treatment groups (*P*=0.95) (**Table 1**).

Additionally, categorical changes in longitudinal LSM according to baseline STE stage (<6.5kPa or ≥ 6.5kPa and < 10.3 or ≥10.3 kPa) are depicted in **Figures 1C, D**. The cumulative incidence of liver fibrosis progression during follow-up was 39% for the STE cutoff of 6.5 kPa and 6% for the STE cutoff of 10.3 kPa. Notably, one in four patients with baseline STE values ≥10.3 kPa exhibited a reduction to <10.3 kPa. Conversely, no patients demonstrated a reduction in LSM from STE ≥6.5 kPa to <6.5 kPa. The progression and regression rates among treatment subgroups are presented in detail in **Supplementary Table S1**. The longitudinal changes of FIB-4 and the progression and regression rates according to a FIB-4 cut-off value of 1.45 were presented in **Supplementary Figure S1**.

### 3.3 The risk factors associated with abnormal Post-treatment STE values

To assess the influence of established treatment modalities and other factors on post-treatment STE outcomes, we conducted logistic regression analyses. The univariate logistic regression analysis identified variables associated with reexamined STE values ≥6.5kPa (**Table 2**). Notably, four variables (body mass index (BMI), height, duration of psoriasis, and baseline STE value) emerged as potential risk factors, all with P values below 0.1. Treatment of IL inhibitors, TNF inhibitors, and MTX did not significantly correlate with the occurrence of abnormal STE values. Similarly, factors such as age, gender, alcohol use, the severity of psoriasis, the existence of PsA, and baseline laboratory parameters had no statistical significance in this context.

**Table 2:** Factors associated with STE value≥6.5 and STE value≥10.3.

Variable	Univariate analysis for STE≥ 6.5		Univariate analysis for STE≥ 10.3	
	OR (95% CI)	*P* Value	OR (95% CI)	*P* Value
Demograohics				
Age	1.03 (0.98-1.09)	0.23	1.02 (0.94-1.10)	0.71
Gender	1.18 (0.30-4.54)	0.81	0.49 (0.08-3.90)	0.44
Height	0.93 (0.85-1.00)	**0.07**	0.94 (0.84-1.05)	0.24
Weight	1.02 (0.97-1.07)	0.53	1.07 (0.99-1.17)	**0.09**
BMI †	1.20 (1.00-1.49)	**0.07**	1.42 (1.11-1.98)	**0.01**
BMI<23 (Underweight and Normal)	0.41 (0.09-1.65)	0.22	0.00 (NA-lnf)	0.99
23≤BMI<27.5 (Overweight)	1.25 (0.41-3.84)	0.69	0.70 (0.12-4.16)	0.69
27.5≤BMI<32.5 (General obesity)	2.30 (0.57-11.67)	0.26	4.75 (0.76-30.18)	**0.08**
Lifestyles				
Smoking status	1.64 (0.53-5.38)	0.40	0.28 (0.01-1.95)	0.27
Drinking status	1.54 (0.48-5.36)	0.48	0.38 (0.02-2.60)	0.39
Psoriasis-related information				
Age of onset	0.99 (0.92-1.05)	0.63	1.02 (0.93-1.13)	0.66
Duration of psoriasis	1.08 (1.01-1.17)	**0.04**	1.00 (0.90-1.11)	1.00
PASI	1.01 (0.94-1.09)	0.78	1.00 (0.87-1.10)	0.97
BSA	1.01 (0.98-1.05)	0.56	1.01 (0.95-1.05)	0.72
PsA	0.69 (0.22-2.14)	0.52	1.56 (0.26-9.23)	0.61
Prior treatment				
Methotrexate	2.49 (0.82-7.96)	0.11	1.83 (0.32-14.17)	0.51
Acitrenin	1.87 (0.61-6.15)	0.28	0.71 (0.09-4.03)	0.71
Phototherapy	1.75 (0.57-5.56)	0.33	0.60 (0.08-3.37)	0.57
Current therapy				
Anti-IL ‡	0.61 (0.19-1.83)	0.38	0.42 (0.05-2.37)	0.34
Anti-TNF §	1.70 (0.50-6.37)	0.41	1.27 (0.16-7.37)	0.80
Methotrexate	1.12 (0.28-4.96)	0.87	2.37 (0.29-14.58)	0.36
The duration of current treatment	0.62 (0.21-1.75)	0.37	0.61 (0.09-3.03)	0.57
Result of STE				
The diagnosis of fatty liver	0.69 (0.18-2.38)	0.56	1.97 (0.28-39.66)	0.55
Baseline STE value	2.60 (1.37-6.30)	**0.02**	1.44 (1.04-2.15)	**0.04**
Baseline laboratory examinations				
Alanine aminotransferase	1.02 (1.00-1.05)	0.14	1.00 (0.97-1.03)	0.76
Aspartate aminotransferase	1.03 (0.99-1.08)	0.22	1.02 (0.96-1.06)	0.46
Albumin	0.87 (0.67-1.10)	0.26	0.98 (0.69-1.45)	0.92
Low Density Lipoprotein	1.44 (0.73-2.98)	0.59	0.64 (0.23-1.72)	0.37
High Density Lipoprotein	1.16 (0.22-6.33)	0.86	0.23 (0.01-3.29)	0.35
Total cholesterol	1.16 (0.67-2.08)	0.59	0.66 (0.26-1.53)	0.35
Triglyceride	0.93 (0.63-1.30)	0.65	0.94 (0.43-1.44)	0.83
Uric acid	1.00 (1.00-1.01)	0.63	1.00 (0.99-1.01)	0.60
γ-glutamyl transpeptadase	1.01 (1.00-1.03)	0.17	0.97 (0.89-1.01)	0.29
Glucose	1.27 (0.51-3.33)	0.60	1.22 (0.29-4.57)	0.77
Hemoglobin	1.01 (0.97-1.04)	0.62	0.97 (0.92-1.01)	0.15
Platelets	1.00 (0.99-1.01)	0.48	1.00 (0.99-1.02)	0.78
White blood cells	1.17 (0.87-1.61)	0.32	0.72 (0.39-1.18)	0.24

^†^The BMI classification is according to the suggestions for Chinese population from WHO. ^‡^Anti-interleukin inhibitors included secukinumab (25 patients), ixekizumab (1 patient), guselkumab (1 patient). ^§^Anti-TNFi included adalimumab (14 patients), Etanercept (1 patient). BMI, body mass index; BSA, body surface area; CI, confidence interval; IL, interleukin; MTX, methotrexate; OR, odds ratio; PASI, Psoriasis Area Severity Index; PsA, psoriatic arthritis; TNF, tumor necrosis factor; STE, sound touch elastography.

The bold values are for P values <0.1.

Given the constraints of the sample size, we limited the variables included in our multivariate logistic regression model to a maximum of four. The baseline STE value and height were excluded to alleviate the confounding influence of collinearity with BMI (**Supplementary Figure S2**). According to previous literature, factors including age, the presence of PsA, and the use of systemic treatments hold significance in association with increased LSMs. Consequently, we incorporated age and PsA into the final multivariate logistic model via a stepwise selection method. The final multivariate logistic model revealed that elevated BMI (OR 1.26, 95% CI 1.04-1.60, *P*=0.031) was a risk factor for post-treatment STE values ≥6.5kPa (**Table 3**).

**Table 3:** Multivariate regression model for STE≥ 6.5 kPa.

Variables	Odds ratio (95% CI)	*P* value
**Psoriasis duration**	1.08 (0.99-1.19)	0.10
**BMI**	1.26 (1.04-1.60)	**0.031**
**Age**	1.00 (0.93-1.07)	0.95
**PsA**	0.31 (0.08-1.11)	0.08

BMI, Body Mass Index; PsA, Psoriatic arthritis; CI, confidence interval.

The bold values are for P values <0.05.

Furthermore, we investigated risk factors for significant liver fibrosis, employing a threshold value of 10.3 kPa. Our univariate analysis confirmed that both BMI (OR 1.42, 95% CI 1.11-1.98, *P*=0.015) and baseline STE value (OR 1.44, 95% CI 1.04-2.15, *P*=0.042) were robust independent risk factors, reinforcing the reliability of our findings (**Table 2**). However, due to the limited number of patients with STE ≥10.3 kPa (n=6), further multivariate analysis was not feasible.

## 4 Discussion

This prospective cohort study contributes to the current understanding of liver health in patients with psoriasis by documenting a significant rise in STE values following systemic treatments, regardless of whether patients received biologics or MTX. Notably, we identified BMI as a key predictor of abnormal post-treatment STE values, proving more influential than medication type, age, gender, or the presence of PsA.

STE, a novel noninvasive ultrasound elastography technique increasingly utilized for diagnosing chronic liver diseases by assessing liver stiffness and associating with fibrosis (18, 21). In our study, we employed STE to monitor LSM changes within the context of psoriatic disease and its treatments, showing a statistically significant increase in overall post-treatment STE values. This finding contrasts with a previous longitudinal cohort study in the general population, which reported no significant differences between baseline and follow-up LSM values (14). Importantly, we observed a cumulative incidence of liver fibrosis progression of 45% across the total cohort over an average follow-up period of 1.16 years, utilizing the STE threshold of 6.5 kPa. Similarly, Mikolasevic et al. also reported a significant fibrosis progression rate of 17.7% over a median follow-up of 1.8 years in subjects with NAFLD (8). The population in these two studies was different, and our longitudinal study captured a more significant LSM progression in patients with psoriasis receiving systemic treatment. Nevertheless, the contributing factors remain elusive yet, and the potential influence of underlying psoriasis and concurrent metabolic conditions cannot be ignored.

Subsequent analyses identified risk factors correlating with abnormal STE values post-treatment. Our findings indicate a significant association between higher BMI and increased susceptibility to liver damage, as suggested by STE values exceeding 6.5 kPa during treatment. This association became more pronounced with a higher STE cutoff level of 10.3 kPa, aligning with previous research linking metabolic factors such as obesity and abdominal adiposity with liver stiffness progression in both the general population and subjects with NAFLD (8, 14, 15). Additionally, our results revealed a linear correlation between baseline STE values and BMI, supporting existing knowledge that NAFLD-related liver fibrosis is associated with metabolic disorders, including overweight and obesity. Previous studies have also documented weight gain among the general population or subjects with NAFLD who suffered increased LSM (14, 15). Thus, we suggested that a high baseline BMI could be a predictor for high follow-up STE value among patients with psoriasis receiving biologics, and we speculated there also some changes in metabolic parameters during follow-up.

Regarding the risk of liver injury associated with systemic treatment, previous studies have explored the impact of MTX usage through liver biopsy and LSM but yielded conflicting conclusions (22, 23). In 2014, a systematic review suggested a potential contribution of MTX to liver fibrosis. Nevertheless, this review included studies with low levels of evidence that failed to control for confounding variables adequately, and the underlying psoriasis and combined metabolic conditions might have influenced the results (23). Conversely, a recent large-scale study showed that the risk of liver fibrosis related to long-term MTX exposure may have been overestimated through LSM (22). Furthermore, the sonographic LSM value changes in patients receiving biologics should be further explored.

Intriguingly, in this study, neither biologics nor MTX was found to be associated with abnormal STE values following systemic therapy, consistent with existing literature that reports no significant differences in serum-based tests for LSM across various therapeutic subgroups, including topical treatments, MTX, and biologics (24). However, our study adds valuable sonographic evidence through the utilization of STE. It suggests that the hepatotoxicity of MTX may have been overestimated, aligning with findings from a recent large multicenter longitudinal cohort study indicating no association between MTX exposure (regardless of cumulative dose or treatment duration) and LSM abnormalities (22). Additionally, we found no conclusive differences in liver stiffness between IL-17 inhibitors and TNF inhibitors.

This study possesses several strengths, including its prospective design and its supplement of dynamic insights into LSM among psoriatic patients undergoing systemic treatment via STE. However, certain limitations must be acknowledged. First, the limited sample size and potential selection bias restricted our analyses to exploratory statistical evaluations. Second, variations in cutoff values for STE may occur based on the population characteristics and specific liver diseases, leaving the diagnostic performance and appropriate thresholds in psoriasis-specific contexts unclear. Third, while we established that the type of systemic therapy is not a risk factor for abnormal post-treatment STE values, we could not evaluate the systemic treatment itself associated risks of hepatotoxicity. Finally, the relatively short intervals between STE assessments and the lack of data regarding hyperlipidemia and diabetes may limit our findings. By addressing these limitations in future research, we hope to gain a more comprehensive understanding of liver health dynamics in psoriatic patients undergoing systemic treatment.

## 5 Conclusions

In summary, our findings indicate no significant association between the type of systemic treatment and abnormal post-treatment STE values. We suggest that MTX may be relatively safe in the absence of comorbidities. Instead, BMI emerges as a principal risk factor and an independent predictor for the development of abnormal post-treatment liver stiffness measurements, particularly at higher STE thresholds. Consequently, we advocate for the management of BMI as an integral component of chronic psoriasis treatment strategies. Future research should engage in large-scale studies that differentiate between subgroups undergoing systemic treatment and those who are not, to elucidate the impact of psoriasis treatments and related metabolic factors on alterations in liver stiffness.

## Data Availability

The raw data supporting the conclusions of this article will be made available by the authors, without undue reservation.
