# Supplementary material for: Systemic treatment type is not associated with abnormal post-treatment noninvasive liver stiffness measurement in psoriasis
Source: Front Immunol. 2024 Dec 11;15:1487959. doi: 10.3389/fimmu.2024.1487959 (PMC11670192; doi:10.3389/fimmu.2024.1487959)
Supplement: Supplementary file 1 [file DataSheet1.docx]

Supplementary figure1. The longitudinal change of FIB-4 value among patients with psoriasis during follow-up.


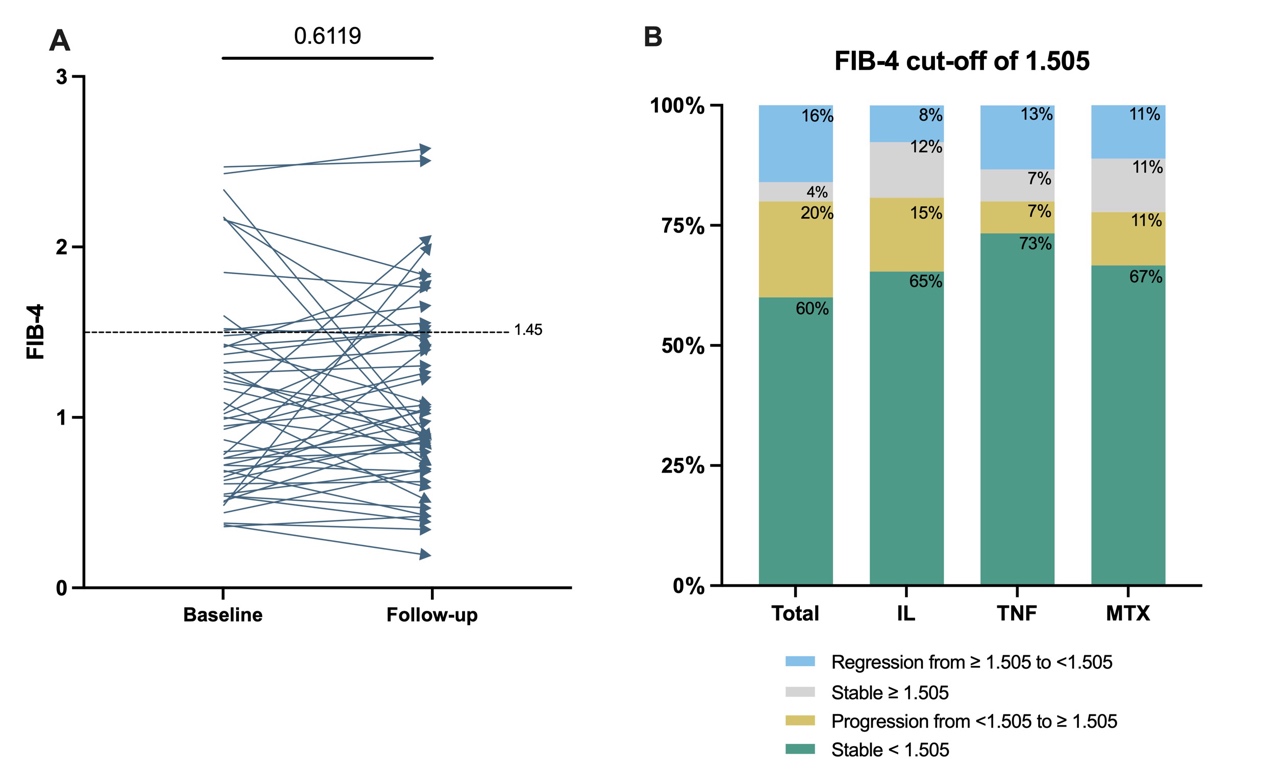


Supplementary figure2. Pearson’s correlation coefficient between continuous variables


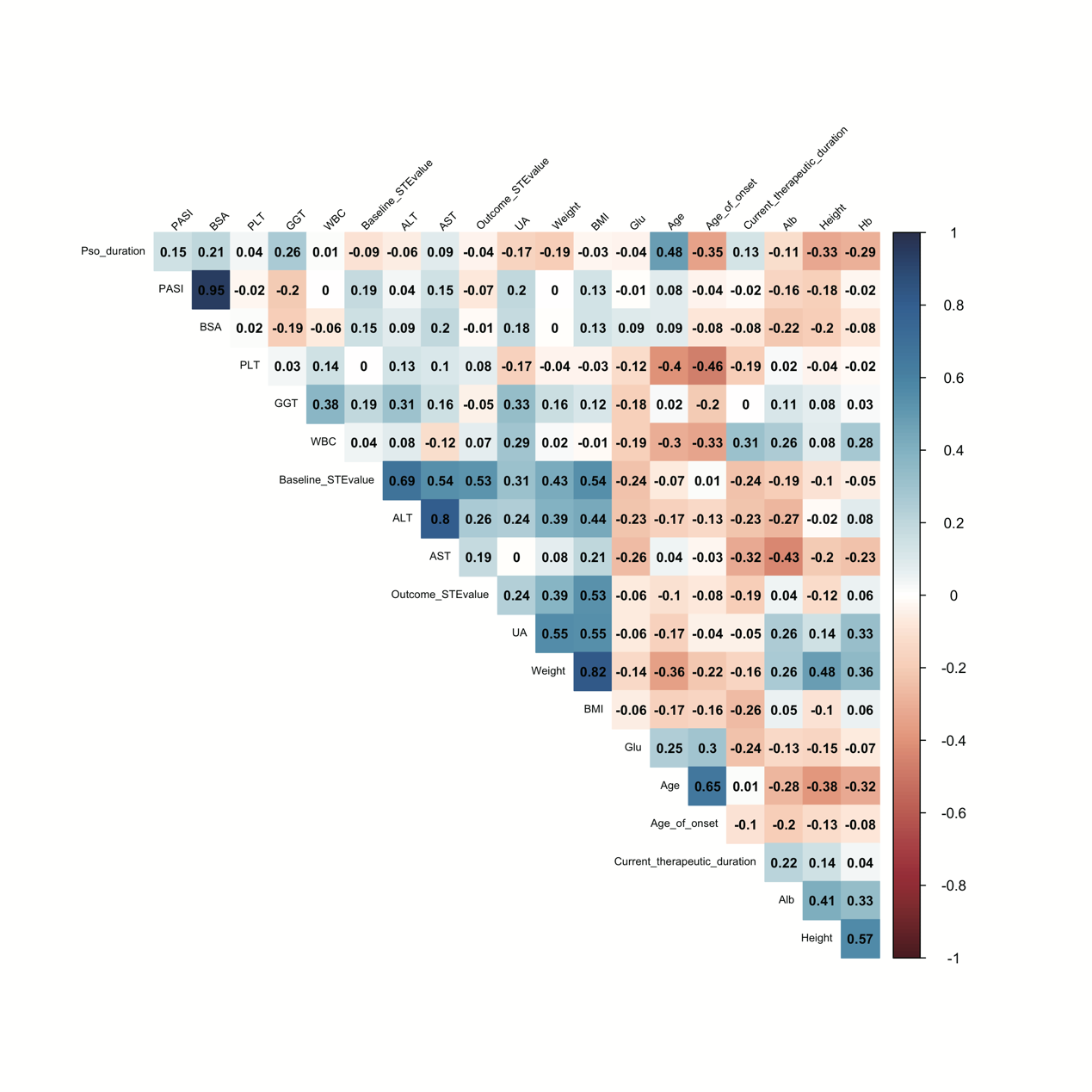


Supplementary table 1. Longitudinal LSM changes by STE cut-off value of 6.5kPa and 10.3kPa

|  | Follow-up | |
| --- | --- | --- |
|  | <6.5 kPa | ≥6.5 kPa |
| <6.5 kPa, n (%) | 24 (55) | 20 (45) |
| (n=44) |  |  |
| ≥6.5 kPa, (n/%) | 0 (0) | 8 (100) |
| (n=8) |  |  |

Total cohort (n=52)

|  | Follow-up | |
| --- | --- | --- |
|  | <10.3 kPa | ≥10.3 kPa |
| <10.3 kPa, n (%) | 45 (94) | 3 (6) |
| (n=48) |  |  |
| ≥10.3 kPa, (n/%) | 1 (25) | 3 (75) |
| (n=4) |  |  |

IL subgroup (n=27)

|  | Follow-up | |
| --- | --- | --- |
|  | <6.5 kPa | ≥6.5 kPa |
| <6.5 kPa, n (%) | 13 (54) | 11 (46) |
| (n=24) |  |  |
| ≥6.5 kPa, (n/%) | 0 (0) | 3 (100) |
| (n=3) |  |  |

|  | Follow-up | |
| --- | --- | --- |
|  | <10.3 kPa | ≥10.3 kPa |
| <10.3 kPa, n (%) | 25 (96) | 1 (4) |
| (n=26) |  |  |
| ≥10.3 kPa, (n/%) | 0 (0) | 1 (100) |
| (n=1) |  |  |

TNF subgroup (n=15)

|  | Follow-up | |
| --- | --- | --- |
|  | <6.5 kPa | ≥6.5 kPa |
| <6.5 kPa, n (%) | 7 (58) | 5 (42) |
| (n=12) |  |  |
| ≥6.5 kPa, (n/%) | 0 (0) | 3 (100) |
| (n=3) |  |  |

|  | Follow-up | |
| --- | --- | --- |
|  | <10.3 kPa | ≥10.3 kPa |
| <10.3 kPa, n (%) | 12 (92) | 1 (8) |
| (n=13) |  |  |
| ≥10.3 kPa, (n/%) | 1 (50) | 1 (50) |
| (n=2) |  |  |

MTX subgroup (n= 10)

|  | Follow-up | |
| --- | --- | --- |
|  | <6.5 kPa | ≥6.5 kPa |
| <6.5 kPa, n (%) | 4 (50) | 4 (50) |
| (n=8) |  |  |
| ≥6.5 kPa, (n/%) | 0 (0) | 0 (0) |
| (n=2) |  |  |

| MTX subgroup | Follow-up | |
| --- | --- | --- |
|  | <10.3 kPa | ≥10.3 kPa |
| <10.3 kPa, n (%) | 8 (89) | 1 (11) |
| (n=9) |  |  |
| ≥10.3 kPa, (n/%) | 0 (0) | 1 (100) |
| (n=1) |  |  |
